# Acute alcohol intoxication and alcohol expectancy effects on women’s memory for consensual and non-consensual sexual activity

**DOI:** 10.3389/fpsyg.2022.1008563

**Published:** 2023-02-01

**Authors:** Laura M. Stevens, Lauren Ann Monds, Benjamin Riordan, Rumandeep K. Hayre, Heather D. Flowe

**Affiliations:** ^1^School of Psychology, University of Birmingham, Birmingham, United Kingdom; ^2^Faculty of Medicine and Health, The University of Sydney, Darlington, NSW, Australia; ^3^Drug and Alcohol Services, Northern Sydney Local Health District, Sydney, NSW, Australia; ^4^School of Psychology and Public Health, Centre for Alcohol Policy Research, La Trobe University, Melbourne, VIC, Australia

**Keywords:** alcohol, alcohol expectancy, memory, rape, sexual assault adult victims

## Abstract

**Objective:**

To test whether acute alcohol intoxication and alcohol expectancy affects how accurately women remember consensual and non-consensual sexual activity that occurred during an interactive hypothetical dating scenario.

**Design:**

A balanced placebo randomized study that varied alcohol dose (mean Breath Alcohol Content; BrAC = 0.06%) and alcohol expectancy prior to participants encoding a hypothetical interactive rape scenario was implemented. Participants could elect to consent to sexual activity with a male partner in the hypothetical scenario. If they stopped consenting, non-consensual sexual intercourse (i.e., rape) was described. Seven days later, participants’ memory for consensual and non-consensual sexual activity in the scenario was tested.

**Main outcome measures:**

Memory accuracy, confidence, and feelings of intoxication.

**Results:**

A total of 90 females (*M* age = 20.5, *SD* = 2.2) were tested regarding their memory accuracy for the consensual and non-consensual sexual activities in the scenario. A multi-level logistic regression predicting memory accuracy for the perpetrator’s behaviors during the rape indicated no effect of alcohol intoxication. However, a main effect of alcohol expectancy was found, whereby participants who expected to consume alcohol, compared to those who did not, recalled the perpetrator’s behaviors during the rape more accurately. A second regression predicting memory accuracy for consensual sexual activity found no main effects for alcohol intoxication or alcohol expectancy. Participants recalled consensual sexual activity with a high degree of accuracy. Calibration analyses indicated that accuracy increased with confidence level, regardless of intoxication level or alcohol expectancy condition, but that women tended to be overconfident in general.

**Conclusion:**

This study provides an important test of how accurately women remember consensual and non-consensual sexual activities. The accuracy of this information is important for forensic medical examinations and police investigations following an allegation of sexual assault. Increased memory accuracy was found for offence details when participants expected to consume alcohol, suggesting there may be important differences in attentional processes (e.g., hypervigilance) depending on whether threat is present. Further research is necessary to investigate memory for sexual violence in real-world settings and to test methods for ascertaining the most complete and reliable accounts.

## Introduction

From 2019 to 2021, 1.6 million adults aged 16–74 years sustained rape or attempted rape in the United Kingdom (Office for National Statistics, 2021). A rape complainant’s statements and testimony are often the primary, if not only evidence, to support an allegation (Tetreault, 1989; Lees, 2002; Kebbell et al., 2007). Whether criminal charges are brought depends on the credibility of the complainant’s account and whether it can be corroborated by other evidence. A key factor affecting credibility in sexual offenses is acute alcohol intoxication on the part of the complainant during the offense. Acute alcohol intoxication is ubiquitous during sexual offenses, and has broad impacts on criminal proceedings, with up to 80% of victims reported to have been alcohol intoxicated when the attack occurred (Mohler-Kuo et al., 2004; Government Equalities Office, 2010).

Acute alcohol intoxication raises questions concerning a witness’s and victim’s ability to accurately remember the crime. Indeed, a prominent survey found that 96% of psychology and law experts believe that witnesses’ memories are less accurate if they were alcohol intoxicated during the crime (Kassin et al., 2001). Further, alcohol use is prevalent not only in sexual offences, but also in consensual sexual encounters. A recent survey found that one third of respondents reported that they or their partner used alcohol during their most recent consensual sexual experience (Herbenick et al., 2019), which in part explains why legal practitioners and jurors are more likely to doubt that the activity was non-consensual when the complainant was under the influence of alcohol (Flowe and Carline, 2021). Moreover, even if law enforcement officials have little doubt that a sexual offense occurred, the complainant’s memory regarding the specific non-consensual activities that happened will be important for purposes of criminal charging (e.g., whether the perpetrator is charged with rape versus sexual assault). To our knowledge, there has been no empirical research on this topic. Therefore, in the current study we explored whether people differentially remember sexual activity, both consensual and non-consensual, if they had consumed alcohol in the lead up to the encounter.

### Theoretical accounts

Anterograde memory impairment, or the marked reduction in the ability to form new episodic memories following acute alcohol intoxication, has been widely demonstrated. Alcohol impairs the ability to consolidate, or form and maintain, new memories by exerting a short-term neurochemical effect on neural networks in the hippocampus. A burgeoning body of eyewitness memory research has examined acute alcohol intoxication and recall accuracy when participant witnesses freely retrieve and report their memories of criminal events. Several studies investigating the effect of intoxication on recall have found that alcohol does not affect how accurately people remember, with these studies analyzing memory performance across a range of violent crimes (e.g., kidnapping, inter-partner violence, and armed robbery) and questioning techniques (Hagsand et al., 2013, 2017; LaRooy et al., 2013; Hildebrand Karlén et al., 2014). A meta-analysis of this literature found that participant witnesses recall fewer correct details about a crime they witnessed whilst alcohol intoxicated compared to sober; however, the number of incorrect details they recall about the crime does not differ depending on alcohol consumption (Jores et al., 2019). Put differently, alcohol seems to affect the completeness, but not the accuracy of memory reports. The consolidation account predicts that people who were alcohol intoxicated compared to sober during a crime will have less complete memories. However, it does not make any clear predictions about alcohol and errors of commission, such as inaccurately recalling during a police interview that a consensual sexual activity was non-consensual. As such, additional theoretical detail is required to make predictions about alcohol and the reporting of erroneous information about sexual activities.

One possibility is that witnesses who were intoxicated during encoding adjust their memory reporting to avoid making errors of commission because of the widespread belief held by laypeople and criminal justice officials that alcohol impairs memory (Flowe and Carline, 2021). The effect of beliefs on memory reporting has been studied in the context of lineup identification. Memory performance can be affected by memory strength manipulations **(**Palmer et al., 2013**)** and alcohol intoxication during encoding (Flowe et al., 2017). Knowledge about eyewitness memory error rates can affect identification performance as well as cued recall performance (Butt et al., 2020). Against this empirical backdrop, intoxicated compared to sober participant witnesses report less information about both consensual and non-consensual activities to offset alcohol-related memory impairment, which in turn reduces the number of errors of commission. Thus, intoxicated and sober individuals will have similar levels of accuracy according to this theory, which we will refer to here as the *alcohol and beliefs about memory account*.

In contrast, the *hypervigilance account* of alcohol and memory reporting predicts that owing to alcohol expectancy effects, memory accuracy for non-consensual activities will be higher if participants believe they have consumed alcohol. The hypervigilance account was originally put forward to explain the observation that attention heightens during risky encounters that involve alcohol. Hypervigilance theory proposes that in situations that have the potential to escalate to sexual assault, people who believe they have consumed alcohol engage in more hypervigilant, or cautious, behavior compared to their counterparts. This behavior arises from the belief that drinking alcohol makes people more vulnerable to sexual victimisation (see Testa et al., 2006). There is some evidence that alcohol expectancies affect memory reports, with women found to remember information more accurately about a hypothetical rape if they thought they had consumed alcohol, suggesting they were more hypervigilant during encoding (e.g., Flowe et al., 2016). Applied to the present study, the hypervigilance account predicts that people who believe they have consumed alcohol will remember non-consensual sexual activities more accurately compared to their counterparts.

A final possibility is that people who are alcohol intoxicated during sexual encounters will be *more* likely to erroneously remember consensual sexual activity as non-consensual. Several studies have examined the impact of alcohol on suggestibility (Schreiber Compo et al., 2012; van Oorsouw et al., 2015, 2019; Gawrylowicz et al., 2017; Evans et al., 2019). Overall, the findings suggest that mere exposure to suggestive information is not sufficient for participants to incorporate the misleading information into memory (Schreiber Compo et al., 2012), including in hypothetical rape encounters (Flowe et al., 2019). However, alcohol intoxication during encoding later renders individuals more susceptible to reporting misinformation if they are repeatedly questioned using inappropriate techniques (e.g., suggestive follow up questions, see van Oorsouw et al., 2015). Nevertheless, perhaps women are more prone to memory distortion when remembering what sexual activities they consented to while alcohol intoxicated, owing to stereotypes about women, alcohol, and sexual availability (see Davis and Loftus, 2015). However, empirical data to support this prediction is completely lacking.

### Current study

In this study we explore the effects of alcohol consumption on how accurately women remember consensual and non-consensual sexual activity. We analyze data from an experiment conducted by Flowe et al. (2019), however, the data have not been reported previously in the literature. If the alcohol and beliefs about memory account is correct, memory accuracy for consensual and non-consensual activities will not vary in relation to alcohol condition. If the hypervigilance account is correct, however, participants who expected to consume alcohol will more accurately remember information about non-consensual activities. Finally, if the memory distortion account is correct, women will less accurately remember information about consensual and non-consensual activities if they had consumed alcohol.

Finally, we also assessed the relationship between confidence and memory accuracy. There is some evidence that women’s confidence in the likely accuracy of their memory for rape is predictive of accuracy, even when they consumed alcohol prior to encoding the rape scenario (Flowe et al., 2017). The present study investigated whether these results extend to memory for consensual and non-consensual activities.

## Method

### Participants

90^1^ female staff and students who were aged between 18 and 32 years (*M* = 20.5, *SD* = 2.2, range: 18–32 years) were recruited from the University of Leicester. Ethics approval for the study was granted by the University of Leicester’s Research Ethics Committee. Written informed consent was obtained prior to participation. Sexual assault largely affects young adult women (Planty et al., 2013), and therefore, the age of the sample was appropriate. For every hour of participation, participants received £4.

### Design

A two beverage (consumed tonic water or alcohol) × two expectancy (told tonic water or alcohol) × two sexual activity (consensual versus non-consensual) mixed design was employed, with sexual activity as the only within subject’s factor. Women were randomly assigned to a beverage and expectancy condition. The outcome measures were memory accuracy, confidence and feelings of intoxication.

A total of 48 women were randomly assigned to the tonic water condition (26 expected alcohol and 22 expected tonic) and 42 to the alcohol condition (22 expected alcohol and 20 expected tonic).

### Materials and procedure

An advertisement for female social drinkers was circulated around campus. Prospective participants took an online pre-screening and were informed that the study was about sexual and dating behaviors and could include discussions about sexual assault.

Women were invited to participate if they scored less than 11 on the Alcohol Use Disorders Identification Test (AUDIT), which was developed by the World Health Organisation to assess harmful, hazardous or dependent drinking (Saunders et al., 1993; Babor et al., 2001). Women who indicated that they did not have any health problems, were not pregnant, and did not use any prescription drugs that would cause an adverse reaction to alcohol were allowed to participate.

Women participated individually in the study. On the day of the study, the participant’s answers on the AUDIT and the general health questionnaire were confirmed by the researcher, and the participant took a urine-based pregnancy test to confirm that she was not pregnant. Participants did not leave the laboratory until their BrAC level was less than 0.02%, and they were asked not to drive an automobile or operate heavy machinery for the rest of the day. Participants were instructed not to consume any alcohol or any food during the 4 h prior to their participation time.

The study then proceeded as follows. We first confirmed that the participant’s BrAC was 0.00% using an AlcoHAWK portable breathalyzer. Next, the participant was given either an alcoholic or a tonic water beverage, depending on the condition to which she had been assigned. In the alcohol beverage condition, the participant was given three cups, each containing five parts tonic water to 1 part vodka to achieve a BrAC of 0.06% (i.e., marginally below the legal driving limit for alcohol intoxication within the United Kingdom). We dosed the participant with alcohol depending on her height and weight, in line with previous research (e.g., Curtin and Fairchild, 2003). In the tonic water beverage condition, women were given 3 cups of tonic water. The cups contained vodka-soaked limes and were rimmed with vodka in both beverage conditions to disguise the alcohol condition to which women had been assigned. Participants consumed each cup within 5 min. In line with other laboratory research, we ensured that the dose of alcohol did not result in a BrAC over 0.06% for ethical reasons.

Half of the participants in each beverage condition were told that they were going to consume alcohol, whereas the other half were told that they were going to only consume tonic water to control for alcohol expectancy. The cups were labelled with “tonic” in the tonic water expectancy condition, and “vodka and tonic” in the alcohol expectancy condition.

The scenario was computer-administered 30 min later after the participant finished her last drink. The participant was told that she would be reading and listening to a scenario about an encounter between her and a man. She was asked to imagine herself in the encounter and to imagine how she would actually think and feel if the encounter actually happened to her. The participant was able to read the text of the scenario as she listened over headphones to it being read aloud by a female.

The scenario was presented *via* the *participant choice* procedure (see Flowe et al., 2007, 2011). This procedure allows the participant to control the activity occurring in the scenario between her and a prospective male dating partner. There were 16 versions of the scenario, which were formed by crossing 4 locations (i.e., bar, her house, his house, and a party) with 4 different types of male dating partner, with each version having unique biographical information about the man (e.g., his occupation, his hobbies, etc). The general plot of the scenario is that the participant has encountered a man at a location, and soon he begins to suggest that he is romantically interested in her. She was instructed that the scenario would unfold one stage at a time, and that at the end of each stage, she would be given a choice about whether to remain in the scenario or to end it (i.e., to tell the man that she wanted to “call it a night”).

If she remained in the entire scenario, consensual sexual intercourse eventually occurred between the participant and the man. In total, there were 22 stages to which the participant could consent, and the final stage was consensual sexual intercourse. The program recorded the stage at which the participant withdrew from the scenario to measure *consent level*.

If the participant called it a night at any stage, a rape continuation scenario was presented. If the participant withdrew before they were alone inside the house, the participant would read that she and the man parted company after she called it a night, but the man later broke into her home, restrained her, said that he would not take “no” for an answer, and had sexual intercourse with her against her will. In other words, a legally definable act of rape was described (Sexual Offences Act, 2003). If the participant withdrew after they were alone inside the house, she reads that the man in the scenario would not take “no” for an answer and restrains her and has non-consensual sexual intercourse with her. Graphic details about the rape were deemed unnecessary to meet the objectives of the study, and therefore, were not provided for ethical reasons. At the conclusion of the scenario, mean BrAC in the tonic water group was 0.00% (*SD* = 0.00), and 0.06% (*SD* = 0.02) in the alcohol group.

The participant was emailed with a link to an online survey 7 days later. The participant was asked to indicate from a list of five sexual activities (kissing, petting above the waist, petting below the waist, oral, sexual intercourse) the activities to which she consensually engaged with the male in the scenario. She also indicated how confident she was in the accuracy of her memory for this information, with the confidence scale anchored from 0% (“just guessing”) to 100% (“extremely sure”).

The survey also contained four multiple choice questions that tested the participant’s memory for details about the events that took place during the rape (e.g., Where did Michael push you? How did Michael hold you down? What did Michael say would happen if you struggled?), all of which included “this did not occur in the scenario” as a response option. The participant also provided a confidence rating (0–100%) regarding how likely it was that her answer was correct for each of these items.

Participants indicated what drink they thought they had consumed (tonic water alone or vodka and tonic), and how intoxicated they felt when reading the scenario using a scale that ranged from 0, “*completely sober*,” to 10, “*completely intoxicated*.”

### Outcome measures

Women’s responses to the consensual sexual activity items were coded for accuracy based on their behavior in the scenario. An item was scored as inaccurate if the participant (1) consented to the sexual activity in the scenario but did not indicate that she did so on the survey, or (2) did not consent to a sexual activity in the scenario but indicated on the survey that she had consented to it.

With respect to the items that tested how well the participant remembered the perpetrator’s behaviors during the rape (non-consensual sexual activity), accuracy was coded based on whether women had read the rape continuation scenario. For participants who did not read it (i.e., they had consented to sexual intercourse), the rape items answered with “this did not occur in the scenario” were scored as correct. If they indicated that a given item occurred, it was scored as incorrect. For women who did not consent to sexual intercourse (i.e., they withdrew from the scenario before consensual sexual intercourse occurred), their answers to these multiple-choice questions were coded for accuracy based on the events described in the rape continuation scenario. *Non-consensual sexual activity confidence* was based on the confidence rating that the participant gave for her responses to the rape activity items.

## Results

### Data analysis plan

Preliminary analyses were conducted to investigate (1) whether between-subjects manipulations influenced participants feelings of intoxication in the experiment using a two (beverage) × two (expectancy) ANOVA; (2) whether participants likelihood to consent differed by between-subjects conditions using chi squared analyses, and (3) whether scenario version influenced dependent variables using a MANOVA. Two separate multi-level logistic regressions were conducted to predict memory for consensual sexual activity and non-consensual sexual activity memory accuracy. Alpha was 0.05 in all analyses.

### Preliminary analyses

First, we examined whether the expectancy and beverage manipulations affected women as intended. A two (beverage) × two (expectancy) between subjects ANOVA indicated that women who consumed alcohol reported feeling more intoxicated compared to those who consumed tonic water [*M* = 5.43, Standard Error of the Mean (*SEM*) = 0.35 vs. *M* = 1.45, *SEM* = 0.33], a significant main effect for beverage, *F*(1,86) = 68.90, *p* < 0.001, *η_p_^2^* = 0.45 (one participant did not answer this question). Women who were told they received alcohol reported feeling similarly intoxicated compared to those who expected tonic water (*M* = 3.74, *SEM* = 0.33 versus *M* = 3.13, *SEM* = 0.35, respectively), a non-significant main effect for expectancy, *F*(1,86) = 1.61, *p* = 0.21; *η_p_^2^* = 0.02. The interaction between beverage and expectancy was also not significant, *F*(1,86) = 1.84, *p* = 0.18; *η_p_^2^* = 0.02.

Overall, 83% (*n* = 75) of participants read the rape scenario continuation (i.e., they “called it a night” at some point in the scenario, and therefore, read the rape depiction). Women who consumed alcohol (*n* = 34) versus those who consumed tonic (*n* = 41) were just as likely to stop consenting and call it a night (81% vs. 85%, respectively), χ2 (1, *N* = 90) = 0.32, *p* = 0.57. Further, women who expected to consume alcohol (*n* = 38) versus those who expected to consume tonic (*n* = 37) were just as likely to stop consenting and call it a night (79% vs. 88%, respectively), χ^2^ (1, *N* = 90) = 1.29, *p* = 0.26. All participants were included in the analysis that followed, even those who consented to sexual intercourse.

According to a MANOVA, the scenario version did not have an effect on any of the dependent variables (Wilks’ Lambda *p* = 0.17), and thus, will not be further considered.

### Alcohol and memory for scenario activities

**Figure 1:**
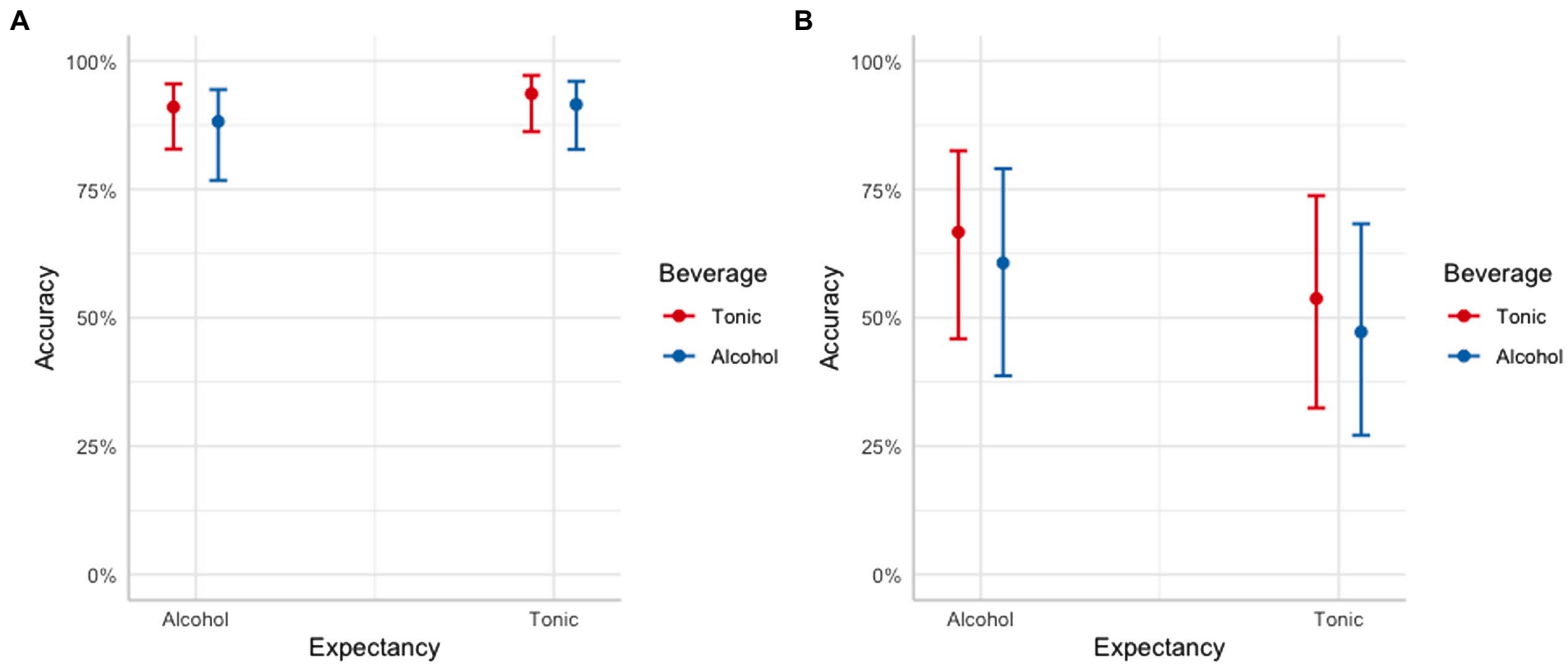
Mean accuracy (±1 SEM) as a function of beverage and expectancy condition for consensual sexual activity **(A)** and non-consensual sexual activity **(B)**.

Figure 1 presents mean memory accuracy (±1 SEM) for consensual sexual activity and non-consensual sexual activity as a function of experimental condition. As can be seen when comparing the data across panels A and B, mean accuracy was greater overall for consensual sexual activities compared to perpetrator’s behaviors during non-consensual sexual activities, regardless of alcohol condition. As reflected by the size of the error bars shown in panel B, there was variability in how accurately participants remembered the perpetrator’s behaviors during the rape, irrespective of condition. In contrast, there was little variability with regards to how accurately participants remembered consensual sexual activities. To explore the effects of alcohol consumption on how accurately women remember consensual and non-consensual sexual activity, we used two separate multi-level logistic regressions.

### Non-consensual sexual activity

We used a multi-level logistic regression which had a binomial (logit link) function as the outcome variable of accuracy is binary (0 = inaccurate, 1 = accurate). At the by-subject level (1|participant), we expected random variation within participants due to individual differences in memory performance. At the by-item level (1|item), we expected random variation across the memory questions arising from item difficulty. We assessed the intraclass correlation for our random intercept model (Accuracy ~1 + (1|participant) + (1|item)) and found that 24% of the variation in this model could be accounted for by the intercept and random effects. This indicates that random effects should be included in the analysis (Raykov, 2011).

The predictor variables were beverage consumed (alcohol vs. tonic), the beverage participants expected to consume (alcohol vs. tonic), self-reported feelings of intoxication, number of days between encoding and memory test, and random effects for participants and items. The analysis was conducted using the *lme4* package (glmer function) in R Software (Bates et al., 2015).

The beverage consumed by participants did not significantly predict their ability to remember non-consensual sexual activities (Wald Z = −0.70, *p* = 0.48, see Table 1 for statistics). Therefore, memory accuracy for non-consensual details did not vary depending on beverage consumed. However, whether a participant expected to consume alcohol, as opposed to tonic water, did predict memory accuracy for non-consensual activities (*B* = −0.54, *SE* = 0.28, Wald Z = −1.97, *p* = 0.049, two-tailed). Participants who expected to consume tonic were less likely to recall accurately the perpetrator’s behaviors during non-consensual sexual activity in comparison to those who expected to consume alcohol (OR = 0.58, 95% CI: [0.34, 1.00]). Feelings of intoxication (Wald Z = −0.43, *p* = 0.69) and number of days between encoding and memory test were not significant (Wald Z = −0.08, *p* = 0.93).

**Table 1:** Multi-level logistic regression analyses predicting memory accuracy for non-consensual sexual activity.

Predictor	B (SE)	*Z*	OR	*95% CI for OR*	
				LL	UL
Intercept	0.84 (0.86)	0.98	2.31	0.43	12.38
Beverage	−0.26 (0.36)	−0.70	0.77	0.38	1.58
Expectancy	−0.54 (0.28)	−1.97*	0.58	0.34	1.00
Feelings of intoxication	−0.03 (0.06)	−0.43	0.97	0.87	1.10
Days	−0.01 (0.11)	−0.08	0.99	0.81	1.22
Variance of random effects					
Participant Item	0.40 0.52				
*Marginal R^2^/Conditional R^2^: 0*.*025/0*.*240^1^*					

*p < 0.05. B, standardized beta value; SE, standard error; Z, Wald Z statistic; OR, odds ratio; LL, lower limit; UL, upper limit. ^1^This was calculated using the method by Nakagawa and Schielzeth (2013).

To assess the power of our model, a simulation-based post-hoc power analysis was conducted using the *simR* package (Green and MacLeod, 2016) in R software (Bates et al., 2015). We ran 1,000 simulations, based on data from our model, to compare our model to the random intercept model using a likelihood ratio test. It was found that our model had 51.90% power (95% CI [48.75, 55.04]) to detect a significant difference, relative to the random intercept model. We also considered power for our expectancy predictor within this model using 1,000 simulations of a Wald Z test. This found 54.70% power (95% CI [51.55, 57.82]) for the expectancy predictor within our model. Additionally, we found a total of 180 participants would be needed to attain over 80% power to detect model effects (95% CI [78.53, 83.48]; Marginal R^2^ = 0.024).

### Consensual sexual activity

A second multi-level logistic regression was computed to predict memory accuracy for consensual sexual activities, using the same predictor variables and random effects as the earlier model. The intraclass correlation for the random intercept model of this analysis showed that 30% of the variance can be explained by random effects at the participant and item level. The main effects for beverage (Wald Z = −0.79, *p* = 0.43), expectancy (Wald Z = 0.92, *p* = 0.36), feelings of intoxication (Wald Z = 0.88, *p* = 0.38), and number of days between encoding and memory test (Wald Z = 0.83, *p* = 0.41) were not significant (see Table 2 for statistics. Thus, whether an individual consumed alcohol or expected to consume alcohol, and how intoxicated they felt did not predict consensual sexual activity accuracy (see Table 3 for descriptive statistics).

**Table 2:** Multi-level logistic regression analyses predicting memory accuracy for consensual sexual activity.

Predictor	B (SE)	*Z*	OR	95% CI for OR	
				LL	UL
Intercept	1.01 (1.37)	0.74	2.75	0.19	40.04
Beverage	−0.38 (0.48)	−0.79	0.68	0.27	1.75
Expectancy	−0.36 (0.39)	0.92	1.43	0.67	3.03
Feelings of intoxication	−0.07 (0.08)	0.88	1.07	0.92	1.26
Days	−0.16 (0.19)	0.83	1.17	0.81	1.69
Variance of random effects					
Participant Item	1.24 0.09				
*Marginal R^2^/Conditional R^2^: 0*.*019/0*.*301^1^*					

B, standardized beta value; SE, standard error; Z, Wald Z statistic; OR, odds ratio; LL, lower limit; UL, upper limit. ^1^This was calculated using the method by Nakagawa and Schielzeth (2013).

**Table 3:** Mean (SEM) for the dependent variables by beverage condition (alcohol consumed versus tonic consumed) and expectancy condition (told alcohol versus told tonic).

	Alcohol consumed		Tonic consumed	
	Told alcohol	Told tonic	Told alcohol	Told tonic
Non-consensual sexual activity accuracy	0.58 (0.06)	0.46 (0.08)	0.64 (0.04)	0.55 (0.02)
Consensual sexual activity accuracy	0.86 (0.04)	0.86 (0.04)	0.84 (0.04)	0.91 (0.04)
Non-consensual sexual activity confidence	68.18 (4.50)	59.75 (5.53)	73.46 (4.43)	65.30 (5.94)
Consensual sexual activity confidence	85.00 (3.21)	92.50 (2.89)	85.77 (3.73)	88.18 (4.64)

We assessed model power using the simulation-based post-hoc power analysis by Green and MacLeod (2016) conducting 1,000 simulations, based on our data, to compare our model to the random intercept model using a likelihood ratio test. This showed that our model had 18.40% power (95% CI [16.04, 20.94]) in finding a difference relative to the random intercept model. Additionally, we found a total of 500 participants would be needed to attain over 80% power to detect model effects (95% CI [81.26, 85.94]; Marginal R^2^ = 0.019).

### Alcohol and the confidence accuracy relationship

**Figure 2:**
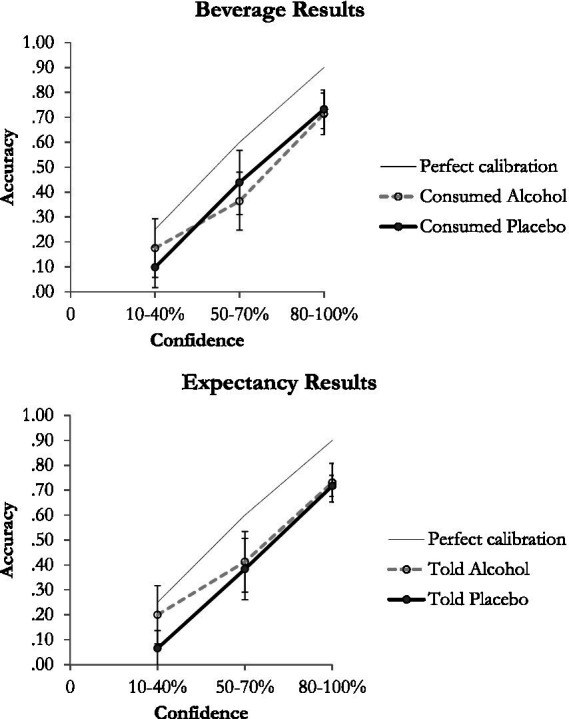
The confidence-accuracy relationship as a function of beverage (top panel) and expectancy (bottom panel). Error bars reflect +/- 1 SEM.

Confidence-accuracy calibration curves as a function of beverage and alcohol expectancy for the non-consensual sexual activity items are presented in Figure 2. To draw the curves, accuracy and confidence data across participants and the 4 items that measured memory for the perpetrator’s behaviors during the rape were pooled, and then average accuracy at each level of confidence was calculated across items. As can be seen, women were similarly calibrated, regardless of whether they had consumed alcohol or were told that they would consume alcohol. As confidence increased, accuracy increased, for women who consumed alcohol and for women who consumed tonic (top panel of Figure 2). This was also the case for women who were told they had received alcohol and for women who were told they had received tonic water (bottom panel of Figure 2). Further, in all conditions, women were overconfident, whereby participants were more confident in their responses than they were accurate.

## Discussion

This study explored whether women who had consumed alcohol (at least up to 0.06% BrAC), or who expected to consume alcohol, compared to their counterparts, differ in their ability to remember consensual sexual activity and non-consensual activities. Women who consumed alcohol during the experiment were just as accurate in remembering consensual and non-consensual sexual activities. Thus, the relationship between remembering sexual activity and alcohol is best accounted for by the alcohol and beliefs about memory account. We also found that participants who expected to consume alcohol were more accurate descriptively speaking (see Figure 1) in remembering non-consensual sexual activities, compared to those who expected to consume tonic, which is in line with the hypervigilance account. Further, regardless of the type of beverage consumed participants were generally overconfident in their memory accuracy. These findings have important implications for practice, which will be discussed next.

Alcohol intoxication affects charging and sentencing in real world cases, with cases involving alcohol being less likely to be prosecuted (Anderson et al., 2007) owing in part to concerns about the credibility and memory accuracy of complainants who were alcohol intoxicated during the offence. These concerns are not borne out by the data of this experiment, at least at the level of intoxication we studied. People appear to be able to remember the activities to which they had consented.

As can be seen in Figure 1A, mean accuracy was not significantly lower in the alcohol compared to the tonic water condition. These findings disconfirm the memory distortion account, which makes the opposite prediction (i.e., that alcohol consumption causes more inaccurate remembering). Further, a post-hoc power analysis indicated that we would need to recruit an additional 410 participants to achieve over 80% power to detect model effects with respect to consensual sexual activities, and an additional 90 participants to achieve over 80% power to detect model effects with regard to the perpetrator’s behaviors during the rape. It is important to bear in mind that accuracy may very well not differ across conditions, and if it does, our data suggest that the size of the effects may be so small as to be of little to no practical importance.

Participants were highly accurate in remembering consensual sexual activities even if they consumed or expected to consume alcohol, with accuracy being greater than 80% in all experimental conditions. Participants also had stronger memories for consensual compared to non-consensual activities regardless of alcohol condition. Participants may have less accurately remembered the actions that took place during the rape for several reasons. First, participants may have been less likely to rehearse these unpleasant, if not traumatic, details, and therefore, information about the non-consensual details were weakly represented in memory. Second, up until the rape was portrayed, participants were engaged throughout the scenario by having to make decisions about whether to consent to the activities being described. In contrast, when they read details about the rape, the details were presented in a passive format, which in turn may have resulted in a weaker memory representation. Third, details about the perpetrator’s actions during the rape (e.g., what the perpetrator specifically said) were presented in fine grain detail, whereas details about the consensual sexual activities that occurred were presented in coarse grain detail (e.g., the participant was merely told that an action happened, like there was kissing, or petting above the waist). Memory for fine compared to coarse grain details is less likely to be accurate (e.g., Butt et al., 2020). We presented the information in the scenario in this manner because the police are more likely to require complainants to report precise details about the nature of the non-consensual activities that happened to determine whether and what sexual offense happened. Detailed information about the nature of the consensual activities is not likely to be probative on the issue of the non-consensual activities being alleged.

Another important consideration is that accuracy may have been higher if participants had reported their memories using free recall rather than closed questions. We used closed questions because previous research indicates that participants seldom freely recall many details about the sexual offense (Larsen et al., 2015; Flowe and Maltby, 2018). However, closed questions can increase the number of inaccurate responses in sober as well as in intoxicated participants (Fisher, 1995; Schreiber Compo et al., 2012; van Oorsouw and Merckelbach, 2012). The use of closed questions can also lead to overconfidence in participants’ judgments about the likely accuracy of their memory (Albright, 2017). In the present study, we found that participants were overconfident, regardless of alcohol condition. The use of open-ended questions can result in more accurate testimony from intoxicated individuals (Schreiber Compo et al., 2012; Altman et al., 2018) and thus should be used to increase recall accuracy (Finn et al., 2019).

An important topic for future investigation is examining how police might better gather detailed information about consensual and non-consensual activities. Victims of rape are often reluctant to provide detailed information about the rape itself, especially if they were alcohol intoxicated during the crime. This is likely because victims of rape are prone to blame themselves for the attack (Janoff-Bulman, 1979), especially if they were alcohol intoxicated (Flowe and Maltby, 2018*)*. Victims may also feel ashamed (Vidal and Petrak, 2007) and may be unwilling to disclose further information for fear of further humiliation and trauma, and negative social judgement (Povey et al., 2009; Relyea and Ullman, 2015). Given these considerations, it is critical to establish and maintain rapport when interviewing victims of sexual violence to help form a trusting relationship which in turn can facilitate the exchange of information (Powell et al., 2005; Ministry of Justice, 2011).

There are several limitations of this study that must be kept in mind with regards to its ecological validity. One limitation is that the participants in this study were of course not exposed to the same degree of trauma as a victim of rape would be. Another limitation is that the alcohol intoxication levels experienced by participants are low to moderate in comparison to alcohol intoxication levels encountered in the field and in intoxicated victims of rape encountered by the police (see Evans et al., 2009; van Oorsouw et al., 2015). Further research is needed under higher levels of intoxication and stress, as well as using more immersive stimuli (e.g., virtual reality). Attention and encoding may very well differ under these circumstances leading to differences in how people remember consensual and non-consensual sexual activities. Having said that, we would predict based on the memory and alcohol beliefs account, that people would report fewer details about consensual and non-consensual activities under conditions where they think that memory might be compromised (e.g., by high levels of intoxication or trauma) to offset possible memory impairment (see Palmer et al., 2013; Flowe et al., 2017). Nevertheless, this study raises important research questions and theoretical avenues in need of investigation, and we hope that this study encourages other researchers to study this important topic.

## Data availability statement

The data analyzed in this study is subject to the following licenses/restrictions: The data may be obtained by contacting the corresponding author. Requests to access these datasets should be directed to h.flowe@bham.ac.uk.

## Ethics statement

The studies involving human participants were reviewed and approved by University of Leicester School of Psychology Ethics Committee. The patients/participants provided their written informed consent to participate in this study.

## Author contributions

HF conceived and designed the research. HF, LS, RH analyzed the data. HF and LS drafted the first version of the manuscript. BR and LM provided theoretical input and contributed to writing the manuscript. All authors contributed to the article and approved the submitted version.

## Funding

The research was funded by the Economic and Social Research Council (ES/J005169/1) to HF.

## Conflict of interest

The authors declare that the research was conducted in the absence of any commercial or financial relationships that could be construed as a potential conflict of interest.

## Publisher’s note

All claims expressed in this article are solely those of the authors and do not necessarily represent those of their affiliated organizations, or those of the publisher, the editors and the reviewers. Any product that may be evaluated in this article, or claim that may be made by its manufacturer, is not guaranteed or endorsed by the publisher.

## Footnotes

^1^We include here the 80 participants who were interviewed (with the Cognitive Interview or Self Administered Interview) in Flowe et al. (2019) as well as 10 pilot participants in that study who were interviewed with the Structured Interview. Note that none of the interview data are being analysed as part of the current research.
